# Multilevel Physiological Responses to the Hypersonic Effect: Concurrent Electroencephalographic, Heart Rate Variability, and Subjective Evaluation in Healthy Adults in a Participant-Blind Crossover Study

**DOI:** 10.7759/cureus.112360

**Published:** 2026-07-09

**Authors:** Ginji Takasao, Satsuki Okanemasa, Tatsuya Kitagawa, Ichika Shimode, Mari Tarumoto, Kanato Miyatake, Takayuki Kodama, Akio Goda

**Affiliations:** 1 Department of Physical Therapy, Faculty of Health and Medical Sciences, Hokuriku University, Kanazawa, JPN; 2 Department of Physical Therapy, Faculty of Health Sciences, Kyoto Tachibana University, Kyoto, JPN

**Keywords:** alpha rhythm, autonomic nervous system, crossover study, electroencephalography, heart rate variability, hypersonic effect, inaudible high-frequency sound

## Abstract

Background and objective

Sounds carrying inaudible high-frequency components (HFCs) above 20 kHz can alter brain activity, autonomic tone, and subjective state, a cluster of responses termed the hypersonic effect. However, only single physiological domains have been examined till now; therefore, whether central, autonomic, and subjective indices move together under one stimulus in the same participants remains unresolved. This study measured electroencephalographic (EEG), heart rate variability (HRV), and subjective responses simultaneously to characterize the layered response to HFC-containing sound.

Methods

Twenty healthy adult men completed a participant-blind crossover protocol in which the order of the two conditions was randomized and counterbalanced (10 received the wide-band sound first, 10 the audible-only sound first): an audible-band sound limited to ≤20 kHz (low frequency (LF)) and a wide-band sound containing the HFC (low frequency plus high frequency (LFHF)). Each condition comprised five minutes of pre-exposure rest, 10 minutes of sound presentation (analyzed as Audio1 and Audio2), and five minutes of post-exposure rest (Post). EEG centro-parieto-occipital (CPO) Alpha-2 relative power, normalized HRV indices, and 20 semantic-differential (SD) ratings were analyzed as changes from the pre-exposure baseline. Aligned rank transform analysis of variance (ART-ANOVA; condition × time) was used, with epoch-specific Wilcoxon signed-rank condition contrasts (Bonferroni correction, m = 3); effect sizes are the rank-biserial correlation (r).

Results

EEG Alpha-2 showed a trend-level main effect of condition (ART-ANOVA F = 3.59, p = 0.074), with no effect of time or condition-by-time interaction. The condition-by-time interaction was not significant; in pre-specified epoch comparisons, the condition contrast was significant during the second half of presentation (Audio2: Bonferroni-corrected p = 0.025, r = 0.657, indicating a large effect) and showed a trend after presentation (Post: corrected p = 0.089, r = 0.552). For HRV, relative low-frequency power (LF%) was significantly lower under LFHF at Post (corrected p = 0.025, r = −0.657, indicating a large effect; median difference −10.1 percentage points, 95% confidence interval −17.0 to −2.9), indicating a reduction in the relative low-frequency component of normalized HRV. None of the 20 SD items differed between conditions (all p > 0.10).

Conclusions

Under these conditions, HFC-containing sound was associated with a trend-level increase in posterior Alpha-2 activity, most evident in pre-specified comparisons during the second half of presentation, and a lower relative LF component of normalized HRV after exposure, without significant differences in subjective ratings. These preliminary findings suggest a possible central-to-autonomic pattern that can be quantified using noninvasive physiological recordings and support further study of HFC-containing sound as an environmental, non-pharmacological approach.

## Introduction

Audible sound spans roughly 20 Hz to 20 kHz, yet many natural sound sources also radiate energy well above this upper limit. These inaudible high-frequency (HF) components (HFCs) are not distributed evenly across everyday acoustic environments. They are rich in certain natural soundscapes, such as tropical rainforests, streams and waterfalls, wind through foliage, and the calls of some insects and birds, and in the sound of a few traditional instruments, such as the Balinese gamelan used in the foundational studies [1]. In contrast, they are largely absent from the environments in which people spend most of their working and resting hours: typical indoor and urban settings radiate little energy above 20 kHz, and conventional recording, broadcasting, and compressed digital-audio formats remove it through band-limiting near 20-22 kHz. As a result, everyday listening, at a desk during work, while commuting, or at rest indoors, rarely contains these components, whereas immersion in an unspoiled natural environment does. The present study reintroduces this normally absent HF energy as a controlled environmental factor to test its physiological effects.

Oohashi et al. reported that sound containing such inaudible HFCs activates deep brain structures and shifts physiological and subjective states, a phenomenon they named the hypersonic effect [1]. In their foundational work, gamelan music was split into an audible component below 22 kHz and an inaudible component above 22 kHz, which produced increased occipital alpha power on electroencephalography (EEG) and elevated regional cerebral blood flow in the brainstem and left thalamus on positron emission tomography (PET), but only when both components were presented together; neither component alone reproduced the effect [1]. Because participants could not consciously hear the HFC, the route by which it reaches the brain has been examined directly. When the HFC was delivered to the ears alone, the effect did not appear, whereas exposure of the whole body surface reproduced it, which points away from conventional air-conduction hearing and toward a mechanosensory pathway whose anatomy is still uncertain [2].

Later studies refined the stimulus conditions. The enhancement of occipital alpha is intensity-dependent and nonlinear, peaking at a modest increment of HFC level [3], and it is frequency-specific: components above approximately 32 kHz raise centro-parieto-occipital Alpha-2 power, while lower HFC bands can reduce it [4]. The time course is also distinctive. The cortical response can emerge gradually rather than instantaneously during presentation and may persist after the sound stops, described respectively as a lagged effect and an after-effect [1,5]. Activation across young and older adults has been reported with combined PET and EEG, suggesting the response is not confined to a single age group [6], and frontal changes have also been described, although in smaller studies [7]. Independent investigators outside the original group have observed that high-resolution audio with inaudible components shifts EEG toward a relaxed attentional state without conscious detection of the difference [8], which lends some external support to the core neural finding.

The hypersonic effect is not limited to cortical rhythms. Sound with HFCs modulates autonomic balance in a context-dependent way, raising both sympathetic and parasympathetic indices during a demanding task while suppressing sympathetic activity during relaxation [9]. High-resolution music delivered through headphones likewise activated heart rate variability (HRV) indices more than a filtered version [10], and HF-amplified music improved parasympathetic recovery after a stressor [11]. Subjective and acoustic-quality effects have been documented since early comparisons of reproduction formats [12]. Together, PET/EEG, body-surface presentation, and frequency-specific EEG findings suggest that deep-brain activation and non-auditory body-surface reception may contribute to these multilevel responses [1,2,4]. The applied interest follows from this breadth. A pilot study explored HFC-containing sound for the behavioral and psychological symptoms of dementia on the rationale that deep-brain activation is a plausible mechanism [13], a single-arm study in terminally ill patients reported reduced anxiety and improved healing scores [14], and music-based interventions already have evidence for reducing agitation in dementia [15].

Two features of this literature define the present gap. First, the central, autonomic, and subjective effects have almost always been measured in separate studies, so it is not known how these layers behave together when one stimulus is presented to one set of participants. A clinical case for environmental sound as a non-pharmacological intervention rests on this kind of integrated evidence rather than on isolated indices. Second, the field carries genuine methodological caveats: reproduction-system artifacts or audible byproducts related to very-HF (VHF) reproduction could contribute to perceived or physiological differences [16], and ultrasound can recruit ascending auditory pathways through cochlear fluid coupling in animal models [17]. A design that records several physiological layers at once and includes a subjective discrimination check addresses part of this skepticism directly, because a physiological change without a perceptible difference between conditions is hard to attribute to expectation alone.

Determining whether HFC-containing sound produces measurable, integrated physiological changes is directly relevant to clinical practice. If a noninvasive environmental stimulus can shift central and autonomic activity toward a more relaxed, parasympathetically weighted state without requiring conscious attention or effort, it could complement existing non-pharmacological approaches in settings where reducing arousal or agitation is a therapeutic goal, such as dementia and palliative care. It may also support stress management and recovery in healthy individuals, including shift workers and athletes. Because this physiological signature can be measured with accessible tools, such as simple EEG and wearable HRV, establishing it is a practical first step toward clinical application and toward identifying individuals most likely to respond.

This study therefore presented an audible-only sound (low frequency (LF)) and a wide-band sound containing the HFC (low frequency plus high frequency (LFHF)) in a participant-blind crossover and recorded EEG, HRV, and subjective ratings simultaneously across the pre-exposure, presentation, and post-exposure periods. We hypothesized that, relative to LF, the LFHF condition would be associated with higher posterior EEG Alpha-2 activity and a lower relative LF component of normalized HRV, while subjective ratings would not differ between the two conditions.

## Materials and methods

Trial design

**Figure 1:**
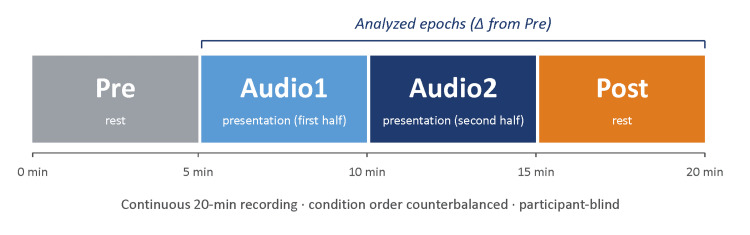
Experimental Timeline Each condition comprised Pre (rest, 0–5 min), Audio1 (presentation, 5–10 min), Audio2 (presentation, 10–15 min), and Post (rest, 15–20 min), recorded continuously over 20 minutes. Audio1, Audio2, and Post were analyzed as change from Pre. Condition order was counterbalanced and participants were blinded.

The study used a two-condition, within-participant crossover design (LF versus LFHF). Both conditions were administered to every participant on the same day, and the presentation order was assigned using computer-generated random numbers (Microsoft Excel RAND function; Microsoft Corporation, Redmond, Washington, United States) and counterbalanced across participants, with 10 receiving LFHF first and 10 receiving LF first. Allocation was generated entirely in software, and participants (but not investigators) were blinded to condition. The two conditions were separated by a 10-minute seated rest interval before the second condition began. Analysis followed the pre-exposure baseline (Pre), early presentation (Audio1), late presentation (Audio2), and post-exposure (Post) structure described below, with all outcomes expressed as change from Pre. Reporting follows the Consolidated Standards of Reporting Trials (CONSORT) extension for crossover trials. The experimental timeline is shown in Figure 1.

The protocol was approved by the Research Ethics Committee of Hokuriku University (approval number 2025-24). All participants received oral and written explanations of the purpose, procedures, and measurements and provided written informed consent.

Participants

Twenty healthy adult men were enrolled and analyzed (mean age 27.4 ± 9.2 years; median 21; range, 21-48 years). Eligibility was restricted to healthy adults. Exclusion criteria were hearing impairment, cardiac disease, psychiatric disease, regular medication use, and sleep disorder.

Sound stimuli and reproduction system

To control the audible and inaudible bands independently, the signal was divided in the digital domain at a 20 kHz crossover and routed to two separate physical output chains. A notebook computer performed playback and signal processing. Audio was played in foobar2000 with Windows Audio Session Application Programming Interface (WASAPI) output (Microsoft Corporation) to deliver the 192 kHz/24-bit source bit-perfectly and to avoid resampling by the operating-system mixer. Signal routing used a virtual audio mixer (VoiceMeeter Banana; VB Audio, Sigoules, France), and band splitting used a system-wide equalizer (Equalizer APO with the Peace front end). A 10th-order Butterworth low-pass filter (cutoff 20 kHz) defined the audible channel, and a 10th-order Butterworth high-pass filter (cutoff 20 kHz) defined the HF channel, producing a steep separation at the crossover.

**Figure 2:**
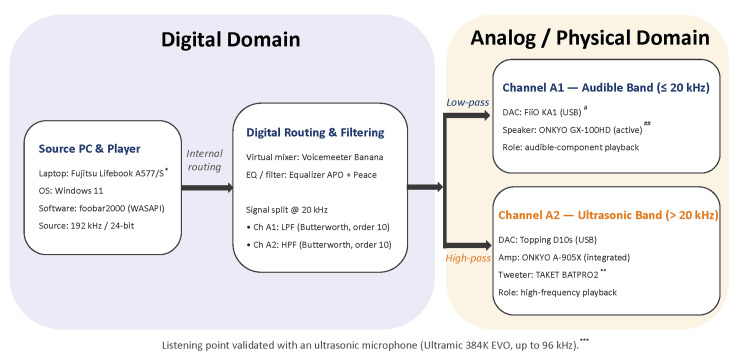
Reproduction System for Audible and HF Channels In the digital domain, the 192 kHz/24-bit source was played bit-perfectly (foobar2000, WASAPI; Microsoft Corporation, Redmond, Washington, United States) and routed through a virtual mixer (VoiceMeeter Banana; VB Audio, Sigoules, France) and a system-wide equalizer (Equalizer APO with Peace), where a 20 kHz crossover split the signal with 10th-order Butterworth filters. The audible channel (≤20 kHz) and the HF channel (>20 kHz) were then reproduced through independent DAC and transducer chains. The radiated spectrum was validated at the listening point with an ultrasonic microphone. ^*^Fujitsu Limited, Nakahara-ku, Kawasaki, Japan; ^##^ Onkyo Corporation, Osaka, Japan; ^**^TakeT Ltd., Kanagawa, Japan; ^#^FiiO Electronics Technology Company, Ltd., Guangzhou, China; *** Dodotronic, Castel Gandolfo (RM), Italy OS, operating system; WASAPI, Windows Audio Session Application Programming Interface; EQ, equalizer; LPF, low-pass filter; HPF, high-pass filter; DAC, digital-to-analog converter; HFC, high-frequency component(s); HF, high-frequency

The audible channel (≤20 kHz) used a Universal Serial Bus (USB) digital-to-analog converter (DAC) (FiiO KA1; FiiO Electronics Technology Company, Ltd., Guangzhou, China) and active loudspeakers (ONKYO GX-100HD; Onkyo Corporation, Osaka, Japan). The HF channel (>20 kHz) used a separate USB DAC (Topping D10s), an integrated amplifier (ONKYO A-905X), and a supertweeter (TAKET BATPRO2; TakeT Ltd., Kanagawa, Japan). The LF condition presented only the audible channel, whereas the LFHF condition presented both the audible and inaudible channels. The source recording was a natural soundscape (“Amazon Rain Forest”) in 192 kHz/24-bit pulse-code modulation (PCM). Loudspeakers and the participant were placed at the vertices of an equilateral triangle with 1.5 m sides, with transducer height set near the participant’s head and the radiating faces directed toward the face. The reproduction system used to separate and present the audible and HF channels is shown in Figure 2.

Acoustic validation

Reproduction level was calibrated in two stages. The audible channel was adjusted with a sound-level meter so that the listening-point level fell within 60-65 dB. The HF channel was tuned to reproduce the spectral balance of the source: sound at the listening point (the participant’s head position) was recorded with an ultrasonic microphone capable of capturing up to 96 kHz (Ultramic 384K EVO; Dodotronic, Castel Gandolfo (RM), Italy) and its spectrum was compared with the source. Supertweeter output was adjusted so that the power balance across the base band (0-20 kHz), HF band (20-40 kHz), and ultra-HF (UHF) band (40-96 kHz) approximated the source as closely as possible, confirming that the HFC reached the listening point through the air.

Outcome measures

Three domains were recorded simultaneously over the full 20-minute protocol.

For EEG, signals were acquired with a multipurpose bioamplifier (Polymate Pro MP6100; Miyuki Giken Co. Ltd., Tokyo, Japan) and an active dry-electrode system, using the international 10-20 system at 19 sites referenced to both earlobes, sampled at 1000 Hz. Signals were band-pass filtered from 1 to 40 Hz, notch filtered at 50 and 60 Hz, and down-sampled to 100 Hz. Independent component analysis (ICA) (extended Infomax, 15 components) was then applied, and components reflecting ocular activity were identified automatically from their correlation with the frontopolar channels (Fp1 and Fp2) and removed; no further automated rejection of noisy channels or epochs was applied. The centro-parieto-occipital (CPO) region of interest comprised seven channels (C3, C4, Pz, T5, T6, O1, and O2). For each channel, the power spectral density was estimated by Welch's method over four-second segments with 50% overlap. The primary EEG outcome was Alpha-2 relative power, defined as the ratio of 10-13 Hz power to 1-40 Hz power, averaged across the CPO channels, expressed as change from Pre (ΔAlpha-2). These preprocessing and spectral-analysis steps, including filtering, ICA-based ocular-artifact rejection, and power spectral density estimation by Welch's method, were performed with MNE-Python (Python 3.10.11; Python Software Foundation, Wilmington, Delaware, United States).

For HRV, R-R intervals were recorded continuously with a chest-worn heart-rate sensor (WHS-3; Union Tool Co., Tokyo, Japan) and analyzed by fast Fourier transform in dedicated software (Kubios HRV Scientific; Kubios Oy, Kuopio, Finland). LF and HF indices in normalized units and as percentages) were computed and expressed as a change from Pre.

For the subjective measure, participants completed a 20-item semantic-differential (SD) questionnaire of adjective-pair impressions on a five-point scale after each measurement, based on the item set used in earlier acoustic-quality work [12]. The SD measure served as an indirect check of whether the two conditions produced perceptible differences in subjective impressions.

Analysis epochs

Recording comprised four consecutive five-minute periods: Pre (rest), Audio1 (first half of presentation), Audio2 (second half of presentation), and Post (rest after presentation). Pre served as the within-condition baseline, and Audio1, Audio2, and Post were analyzed as change from Pre.

Statistical analysis

Because the change scores were not assumed to be normally distributed, a nonparametric factorial approach was used. The effects of condition (LF, LFHF) and time (Audio1, Audio2, Post) and their interaction were tested by aligned rank transform analysis of variance (ART-ANOVA) for within-participant designs. Condition contrasts at each epoch (Audio1, Audio2, and Post) were pre-specified in the study protocol as a priori comparisons and were therefore conducted independently of the omnibus ART-ANOVA result; they were tested with Wilcoxon signed-rank tests, with Bonferroni correction applied across the three pre-specified epoch comparisons (m = 3). Direct LF-versus-LFHF comparisons of SD ratings used the Wilcoxon signed-rank test.

The effect-size measure for the Wilcoxon tests was the matched-pairs rank-biserial correlation (r), interpreted as small (0.10), medium (0.20), large (0.30), and very large (0.40) [18], and supplemented for the principal contrasts by the Hodges-Lehmann median difference with a distribution-free 95% confidence interval (CI). The significance level was set at α = 0.05 (two-sided), and 0.05 ≤ p < 0.10 was reported as a statistical trend. Established conventions guided the interpretation of effect magnitude [18]. The primary statistical analyses - the aligned rank transform ANOVA and the pre-specified Wilcoxon signed-rank condition contrasts - were performed in Python (version 3.10.11) using the pingouin (version 0.6.0) and SciPy (version 1.15.3) packages.

## Results

A total of 20 participants were included in this study and analyzed, with the presentation order counterbalanced (10 received LFHF first and 10 received LF first).

EEG: posterior Alpha-2 activity

**Figure 3:**
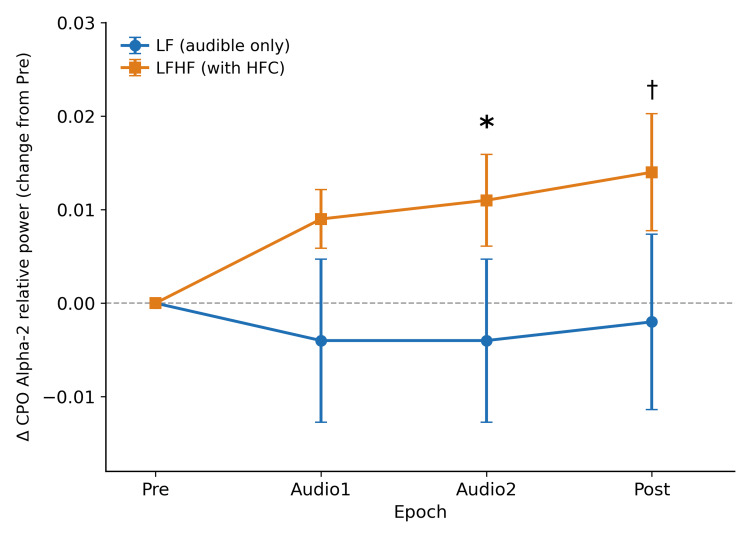
CPO Alpha-2 relative power by condition Time course of CPO Alpha-2 relative power, expressed as change from Pre, across Pre, Audio1, Audio2, and Post. Points are group means and error bars are standard error (n = 20). *Bonferroni-corrected p = 0.025 at Audio2 (r = 0.657); †trend (p = 0.089) at Post (r = 0.552). CPO, centro-parieto-occipital; LF, low frequency (audible-only condition) (≤20 kHz); LFHF, low frequency and high frequency (wide-band condition containing the HFC); r, rank-biserial correlation; Pre/Audio1/Audio2/Post, analysis epochs.

ART-ANOVA of CPO ΔAlpha-2 showed a trend-level main effect of condition (F = 3.59, p = 0.074). The main effect of time (F = 0.37, p = 0.696) and the condition-by-time interaction (F = 0.01, p = 0.988) were not significant. Convergent analyses of the condition difference agreed with this trend (repeated-measures ANOVA p = 0.061; Wilcoxon signed-rank test on the condition means collapsed over epochs p = 0.064, r = 0.476, median difference 0.010, 95% CI −0.0004 to 0.026). Across all epochs, ΔAlpha-2 was higher under LFHF than under LF (Table 1). The time course of CPO ΔAlpha-2 by condition is shown in Figure 3.

**Table 1:** CPO Alpha-2 relative power (change from Pre): descriptive statistics and pre-specified condition contrasts (n = 20) Values have changed from Pre. Median difference is the Hodges-Lehmann estimate (LFHF − LF) with distribution-free 95% CI. ART-ANOVA results are reported in the main text. CPO, centro-parieto-occipital; LF, low frequency (audible-only condition) (≤20 kHz); LFHF, low frequency and high frequency (wide-band condition containing the HFC); SD, standard deviation; CI, confidence interval; ART-ANOVA, aligned rank transform analysis of variance; r, rank-biserial correlation.

Epoch	LF (mean ± SD)	LFHF (mean ± SD)	p (uncorrected)	p (Bonferroni)	r	Median difference (95% CI)
Audio1	−0.004 ± 0.039	0.009 ± 0.014	0.177	0.531	0.352	0.009 (−0.003 to 0.027)
Audio2	−0.004 ± 0.039	0.011 ± 0.022	0.008	0.025	0.657	0.012 (0.003 to 0.024)
Post	−0.002 ± 0.042	0.014 ± 0.028	0.030	0.089	0.552	0.010 (0.002 to 0.033)

The epoch contrasts placed the difference in the second half of the presentation. The conditions did not differ during early presentation (Audio1: LF −0.004 ± 0.039, LFHF 0.009 ± 0.014; corrected p = 0.531, r = 0.352). The difference was significant during late presentation (Audio2: LF −0.004 ± 0.039, LFHF 0.011 ± 0.022; uncorrected p = 0.008, corrected p = 0.025, r = 0.657, indicating a large effect; median difference 0.012, 95% CI 0.003 to 0.024) and showed a trend after presentation (Post: LF −0.002 ± 0.042, LFHF 0.014 ± 0.028; uncorrected p = 0.030, corrected p = 0.089, r = 0.552; median difference 0.010, 95% CI 0.002 to 0.033). Because the condition-by-time interaction was not significant, the magnitude of the condition difference did not vary statistically across epochs; within the pre-specified epoch comparisons, however, the difference reached significance at Audio2 and a trend at Post. This pattern should therefore be read as a consistent condition-level difference at the trend level, with the pre-specified comparisons locating the clearest effect in the late presentation window.

HRV: autonomic balance

**Figure 4:**
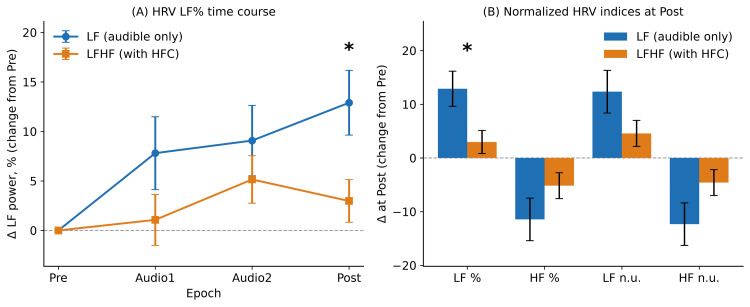
Normalized heart rate variability indices by condition Normalized HRV indices expressed as change from Pre. (A) LF% time course across epochs (mean ± SE, n = 20). (B) The four normalized indices at the Post epoch (mean ± SE, n = 20). *LF% Bonferroni-corrected p = 0.025 (r = −0.657), indicating lower relative low-frequency power under LFHF. HRV, heart rate variability; LF%/HF%, low-/high-frequency power as a percentage of total power; LF n.u./HF n.u., low-/high-frequency power in normalized units; LFHF, low frequency and high frequency (wide-band condition containing the HFC); SE, standard error; Pre/Audio1/Audio2/Post, analysis epochs. Note: the "LF/LFHF" in the legend keys refer to conditions; LF%/HF% refer to HRV bands

For the normalized HRV indices, none of the condition main effects reached significance (LF% p = 0.120; HF% p = 0.664; LF and HF in normalized units p = 0.530 and p = 0.531). The main effect of time was significant, or trend-level across indices (HF in normalized units p = 0.049; LF in normalized units p = 0.050; HF% p = 0.063; LF% p = 0.068), and no interaction was significant (Table 2). At the post-exposure epoch, the relative LF index (LF%) was significantly lower under LFHF than under LF (uncorrected p = 0.008, corrected p = 0.025, r = −0.657, indicating a large effect; median difference −10.1 percentage points, 95% CI −17.0 to −2.9). The corresponding contrasts for the other normalized indices at Post were not significant after the Bonferroni correction. The lower LF% under LFHF at Post indicates a shift in normalized autonomic balance, with a reduction in the relative LF component after exposure to the HFC-containing sound (Figure 4).

**Table 2:** Normalized HRV indices (change from Pre): ART-ANOVA and Post-epoch contrasts (n = 20) Condition, Time, and Interaction columns are ART-ANOVA results; Post columns are Wilcoxon signed-rank contrasts (LF vs LFHF) at Post with Bonferroni correction. n.u., normalized units; n.s., not significant. For LF (%), the Hodges-Lehmann median difference (LFHF − LF) was −10.1 percentage points (95% CI −17.0 to −2.9). HRV, heart rate variability; LF, low-frequency HRV band; HF, high-frequency HRV band; n.u., normalized units; ART-ANOVA, aligned rank transform analysis of variance; n.s., not significant; r, rank-biserial correlation; Post, post-exposure epoch. Note: here LF and HF denote HRV spectral bands, not the experimental conditions (LF/LFHF)

Index	Condition (p)	Time (p)	Interaction	Post: p (Bonferroni)	Post: r	Post: result
HF (n.u.)	0.531	0.049	n.s.	0.292	+0.429	n.s.
LF (n.u.)	0.530	0.050	n.s.	0.292	−0.429	n.s.
HF (%)	0.664	0.063	n.s.	0.607	+0.333	n.s.
LF (%)	0.120	0.068	n.s.	0.025	−0.657	Significant

Subjective ratings

None of the 20 SD items differed significantly between conditions (all p > 0.10; smallest p = 0.130 for variation in nuance). Condition differences in mean ratings were small throughout, ranging from −0.25 to +0.40 scale points (Table 3). The SD ratings did not reveal significant subjective differences between the LF and LFHF conditions, which is consistent with maintained blinding and suggests that the physiological differences are not readily explained by a perceived difference between the conditions.

**Table 3:** Semantic-differential ratings by condition (n = 20). English labels are translations of the original Japanese adjective pairs. Five-point scale was used; all 20 items LF, low frequency (audible-only condition) (≤20 kHz); LFHF, low frequency and high frequency (wide-band condition containing the HFC); SD, semantic differential; Diff, mean difference (LFHF − LF).

Item (low–high anchor)	LF	LFHF	Diff	p
Harsh – pleasant resonance	4.05	4.05	0.00	1.000
Hard – soft	4.20	4.05	−0.15	0.366
Small – large variation in nuance	3.30	3.70	+0.40	0.130
Thin – thick	3.35	3.30	−0.05	0.869
Artificial – natural	4.40	4.50	+0.10	0.317
Shallow – deep	4.35	4.40	+0.05	0.763
Dry – moist	4.50	4.55	+0.05	0.705
Attack-type – reverberant	3.90	4.05	+0.15	0.396
Sparse – rich in information	4.40	4.45	+0.05	1.000
Tiring – not tiring	4.10	4.15	+0.05	0.958
One instrument prominent – balanced	3.10	3.10	0.00	1.000
Lively – quiet	2.80	2.75	−0.05	1.000
Heavy – light	3.60	3.35	−0.25	0.197
Lacking – having realism	4.20	4.25	+0.05	0.705
Coarse – fine texture	3.65	3.85	+0.20	0.405
Narrow – wide spatial spread	4.45	4.50	+0.05	0.317
Poor – rich atmosphere	4.40	4.60	+0.20	0.157
Tense – relaxed	4.45	4.40	−0.05	0.763
Dislike – like	4.25	4.15	−0.10	0.480
High-tone – low-tone prominent	2.40	2.20	−0.20	0.257

## Discussion

Presenting the audible-only and HFC-containing sounds to the same participants while recording three physiological layers at once produced a coherent picture: posterior Alpha-2 activity was higher under the HFC-containing sound, the relative LF HRV index was lower after that sound, and subjective ratings did not separate the conditions. Collectively, these results describe a response that reaches both the central and autonomic levels, yet was not accompanied by significant differences in subjective ratings. The dissociation matters for interpretation because a physiological shift that is not reflected in subjective ratings is less readily attributable to expectation, and it is the kind of integrated evidence that an isolated single-domain study cannot provide.

The EEG finding fits the established account of where alpha rhythms are generated and modulated. Posterior Alpha-2 is associated with relaxed, internally directed states, and the hypersonic literature locates the driver of HFC-related alpha enhancement in brainstem and thalamic activation that propagates to cortex [1,4]. This is consistent with independent neuroimaging work in which alpha power correlates with thalamic and midbrain activity rather than arising in cortex alone [19-21], and with a meta-analytic synthesis showing that alpha tracks relaxation in a topographically specific manner [22]. The difference was clearest in the second half of the presentation, where the effect size was large, which is in line with the lagged effect described for HFC-containing sound, in which the cortical response builds over minutes [1,5,8]. We frame the broader claim cautiously, because the omnibus main effect of condition reached only a trend and the interaction was not significant; the clearest statistical finding is the large condition difference at the late presentation epoch, with a weaker trend afterward.

The autonomic result is directionally consistent with the wider literature. A lower LF% after exposure indicates a reduction in the relative LF component of normalized HRV, a direction broadly consistent with previous reports on HFC-containing and high-resolution sound at rest and on HF-amplified music during stress recovery [9-11], and with reviews showing that music reliably increases parasympathetic activity [23]. In normalized HRV, a lower relative LF component may be cautiously interpreted as a shift toward parasympathetic predominance, or equivalently a reduction in relative sympathetic outflow, although the LF band reflects both sympathetic and parasympathetic influences and should not be read as a pure index of sympathetic activity. The absence of condition main effects for the HRV indices, together with a significant contrast only at the post-exposure epoch, suggests that any autonomic change is small and emerges late rather than during presentation. This pattern of an earlier and clearer central effect alongside a later and subtler autonomic effect is compatible with, but does not prove, a central-to-autonomic sequence; the present data establish association at specific epochs rather than a tested mediation pathway.

The subjective results carry interpretive weight beyond their null value. Because the SD ratings did not differ significantly between conditions on any of the 20 items, the EEG and HRV differences are unlikely to reflect a conscious preference or demand effect. This speaks to a standing concern in the field, namely whether reproduction-system artifacts or audible byproducts related to very-high-frequency reproduction could contribute to physiological differences [16,24], and whether ultrasound might recruit auditory pathways indirectly [17]. Independent control of the two bands through separate transducers, validation of the radiated spectrum at the listening point with an ultrasonic microphone, and a subjective discrimination check together reduce, though they do not eliminate, these alternative explanations.

Several limitations qualify these conclusions. First, the sample of 20 limited statistical power; the EEG condition main effect reached only a trend, and the time-resolved interpretation rests on a single significant epoch contrast rather than on a significant interaction. Second, the HRV evidence is similarly confined to one significant epoch contrast without a supporting condition main effect, so it should be treated as preliminary. Third, the sample was restricted to healthy adult men, so the response may differ by age, in women, or in clinical populations, none of which this design could examine. Fourth, the study did not relate the physiological changes to individual subjective ratings, so the link between perceived state and measured physiology remains open [15]. Fifth, much of the hypersonic-effect literature originates from a single research group; independent replication remains limited, and the artifact and cochlear-pathway questions are not fully resolved [16,17]. Sixth, because the two conditions were presented on the same day, separated by a 10-minute rest interval, and order and carryover effects were not modeled statistically, residual carryover cannot be fully excluded despite counterbalancing of presentation order. The EEG preprocessing relied on ICA-based removal of ocular components without additional automated rejection of noisy channels or epochs, which may have left residual muscle or movement artifacts; future work should incorporate more comprehensive artifact-handling procedures. While this dissociation suggests that the physiological shifts are not merely driven by conscious expectation or demand characteristics, the absence of subjective differences might equally imply a very subtle overall stimulus effect; these epoch-specific physiological changes must therefore be interpreted with caution until confirmed by larger replications. Finally, only a short, single exposure was tested, so cumulative or longer-duration effects are unknown.

Within these limits, the practical implication is concrete. Simple EEG with independent component analysis and a region-of-interest reduction, combined with wearable HRV, was sufficient to detect a central and an autonomic signature of HFC-containing sound. Because the stimulus is noninvasive, requires no conscious attention, and can be layered over ordinary audible sound, it is a plausible candidate for environmental, non-pharmacological support of relaxation and autonomic balance, with possible relevance to settings such as dementia care, where music-based approaches already show benefit [13-15]. Confirmation will require larger samples, clinical populations, longer and repeated exposures, and analyses of responder characteristics. From a clinical-practice standpoint, these findings point to a low-burden adjunct rather than a stand-alone treatment: because the stimulus can be layered over ordinary audible sound and requires no active participation, it could be delivered passively in care environments or during rest and recovery for workers and athletes. Two considerations bear on safety and the prevention of harm. First, the audible channel was held at a moderate level (60-65 dB), and the HF channel was power-balanced to a natural soundscape rather than amplified, so exposure approximated that of a natural acoustic environment. Second, although adverse symptoms have been reported for high-level audible VHF sound and for intense ultrasound [16,17], the levels used here were well below those contexts; any applied use should nonetheless monitor exposure level and duration, particularly in vulnerable populations. These points delineate both the potential benefit, supporting relaxation and autonomic balance, and the precautions needed to avoid harm to patients, workers, or athletes.

## Conclusions

In this participant-blind crossover study, sound containing inaudible HFCs was associated with a trend-level increase in posterior Alpha-2 EEG activity, most evident in pre-specified comparisons during the second half of presentation, and a lower relative LF component of normalized HRV after exposure, without significant differences in subjective ratings. Simultaneous recordings across the central, autonomic, and subjective domains allowed quantification of these layered responses using accessible physiological tools. These preliminary findings support continued study of HFC-containing sound as a noninvasive environmental approach and point to larger, clinically oriented trials with longer and repeated exposures as the next step.
